# Hearne Is Ahead

**Published:** 1899-07

**Authors:** Thos. J. Pugh

**Affiliations:** Hearne, Texas


### Correspondence.

### Hearne Is Ahead.

HEARNE, TEXAS, June 11, 1899.

On the night of June 7, one M. C. B., a druggist of Hearne, concluded that he would castrate himself. With that end in view, he proceeded to do the operation on a quick and non-expensive, non-aseptic plan. He applied cocaine solution to his scrotum till all sensibility of the parts was destroyed, and with a common tobacco pocket-knife opened the scrotal sack and took out his testicles, severed the cord, and put the organs away in a pickle bottle, and has them preserved in alcohol. The loss of blood was great, but the operator controlled his hemorrhage with ice cold compresses. The patient was found Thursday morning, about 9 o'clock. He was almost exsanguinated, and after much persuasion he permitted a physician to see and stitch the wound he had self-inflicted.

He talks sensibly about the matter, and says he has contemplated the act for several years. The patient has never suffered with much pain, and will not take any medicine. He says if he had known the operation could have been so easily done he "would have removed his balls ten years ago." He sits up and reads the daily papers as though nothing unusual had happened to him.

The success of this operation, without any regard to the "aseptic regime," certainly knocks out that fad.

THOS. J. PUGH, M. D.

